# Architecture and Behavior-State Classification of a WebSocket-Based IMU Stream Extraction Workflow for Pico 4 Headsets

**DOI:** 10.3390/s26196151

**Published:** 2026-09-28

**Authors:** Ruimeng Li, Zhixin Cai, Siyuan Song, Mingxuan Cai, You-Lei Fu

**Affiliations:** 1 School of Design and Fashion, Zhejiang University of Science and Technology, Hangzhou 310023, China; 2 Interactive Media Institute, Academy of Arts & Design, Tsinghua University, Beijing 100190, China

**Keywords:** Pico 4 Pro, inertial sensing, ADB, WebXR, DeviceMotion, time-based window, data quality, reproducibility

## Abstract

**Highlights:**

- Thirty exports from 34 participants yield 29 retained sessions for analysis.
- Post hoc timing groups are kept separate because acquisition-source metadata are absent.
- Five accMag series and 28 Euler-field sets are flagged as session-wide constant.
- Matched window comparisons and channel audits quantify changes in deterministic rule outputs.

**Abstract:**

Motion exports from consumer virtual-reality headsets may obscure measurement semantics and acquisition timing. We audited Pico 4 Pro exports from 34 participants, each participating once. Thirty CSVs were available; excluding an 8.410 s export left 29 sessions and 45,560 samples. Source labels, raw axes, and hardware timestamps were absent. Post hoc timing groups comprised 27 ADB-like files (median 3.745 Hz) and two browser-compatible files (8.333 and 20.000 Hz), without confirming acquisition routes. Five acceleration-magnitude series and 28 Euler-field sets were constant. Reanalysis used trailing windows of up to 2.0 s, long-gap resets, and channel gates. A reset-matched 21-sample comparator changed 2.428% of ADB-like outputs and 0.864% in the two higher-rate files. Two raters supplied 661 paired one-second bins covering 2400 samples from ten selected sessions. Inter-rater kappa was 0.698 (session-cluster 95% CI 0.615–0.777). Agreement between a separate three-group gyro-based output and the raters were 0.530 (0.275–0.657) and 0.529 (0.317–0.618). The audit makes unavailable inputs, denominators, and configuration dependence explicit. It supports reproducible export-level analysis, not sensor accuracy or validated behavioral classification.

## 1. Introduction

Consumer virtual-reality headsets combine rendering, inside-out tracking, inertial sensing, and runtime pose estimation. Headset tracking has been evaluated against external motion-capture systems [1], and head orientation has been studied under controlled display-lag conditions [2]. Other work has examined postural stability during virtual-reality exposure [3]. A device-level comparison including Meta Quest 3 and Pico 4 Pro likewise evaluates tracking rather than assuming measurement equivalence from interface names [4]. These uses motivate a prior question for secondary analysis: what measurement and timing information actually survives in an exported file?

Wearable and smartphone studies show that segmentation and feature representation affect activity analysis [5,6,7], including seated behaviors [8,9] and measurements from different body locations [10]. Supervised examples use deep time-frequency models [11], explicit training and cross-validation [12], and sample-level data valuation [13]. The present archive instead contains mapped magnitudes, exported orientation fields, and platform-generated labels, with no laboratory reference or held-out evaluation set. A limited third-person video subset provides an agreement reference, not comprehensive behavioral ground truth.

Open systems address different layers of this problem. The Unity Experiment Framework supports VR experiment organization and application-level data files [14]. OpenSense makes calibrated IMU orientations, biomechanical modeling, and optical comparison explicit [15]. Lab Streaming Layer (https://labstreaminglayer.org/; accessed on 16 September 2026) synchronizes software streams using sample timestamps and clock-offset and jitter correction [16]. WebXR returns poses at XRFrame times [17]; OpenXR locates space poses at historical or predicted times [18]; DeviceMotion defines browser motion events [19]; Android distinguishes hardware and software sensors [20]; and ADB supports command-line device communication [21]. These interfaces are not interchangeable measurement sources. Table 1 compares their roles with the present audit.

Data quality is itself a requirement before activity classification [22]. Fusion implementations differ in estimation and computational properties [23], optical comparison requires coordinate-frame alignment [24], and joint-angle estimation depends on calibration and anatomical alignment [25]. Low-cost systems also involve filtering and calibration assumptions [26,27] and practical deployment constraints [28,29]. A review in people living with spasticity illustrates heterogeneous reporting within that application [30]. Alignment, data loss, and noise affect sensor-data analysis [31]; drift is a further concern in single-IMU displacement estimation, including quasi-cyclical applications [32]. Optical references can support validation, but their own positioning performance must be characterized [33,34].

This study asks four questions: RQ1, what timing, field-semantic, and channel-quality differences are visible in the exports? RQ2, how does a 2.0 s window differ from a 21-sample history when gap handling is held constant? RQ3, how sensitive are outputs to window duration, thresholds, priority order, and Euler-range implementation? RQ4, what agreement is observed in the selected two-rater video subset? We contribute a traceable field-and-timestamp audit, channel-eligible denominators, controlled configuration comparisons, and a documented annotation subset. These are reusable analysis steps, not a new sensor interface or validated behavioral classifier. Samples are repeated observations within sessions, which are the analysis units.

**Table 1:** Roles of related workflows and laboratory reference systems. This is a functional comparison, not a performance benchmark.

Workflow	Data Layer	Timing, Calibration, and Deployment	Role Relative to This Audit
Present Pico export audit	Mapped magnitudes, Euler fields, export timestamps, rule outputs	Headset plus host; no retained raw axes, hardware clock, or calibration record	Retrospective export-level checks
Unity Experiment Framework [14]	Experiment structure; application data	Unity project and VR hardware; application timestamps and file output	Open VR experiment organization
OpenSense [15]	IMU orientations and musculoskeletal kinematics	Body-mounted IMUs; calibration and fusion; optical comparison	Calibrated IMU-processing comparator
Lab Streaming Layer [16]	Networked multimodal streams	Clock-offset and jitter correction between timestamped streams	Software-stream synchronization
Optical motion capture [24,33,34]	External marker pose	Cameras, calibrated space, markers, and frame alignment	External reference for future validation

## 2. Materials and Methods

### 2.1. Study Context, Device, and Record Flow

A Pico 4 Pro headset was used during seated 360-degree video viewing. Dated exports and reports span 21–24 April 2026. Thirty-four distinct participants provided informed consent, and each participated once. Identifiers S001–S034 link the consent inventory, reports, and available exports. The retained session is the analysis unit and corresponds to one participant.

The inventory contains 30 analysis CSV exports and 36 dated reports (20 HTML and 16 PDF). S001 and S002 have multiple reports, while S012 has a PDF report but no analysis CSV. The retrospective completeness criterion was a first-to-last exported span of at least 60 s. S002 contained 842 rows over 8.410 s and was excluded; the shortest retained file spanned 122 s. S003, S004, S012, and S016 had no analysis CSV. Thus, 29 sessions were retained: S001, S005–S011, S013–S015, and S017–S034. Export 1776846533989 maps to S013 and export 1776847632327 to S014. Table 2 distinguishes exclusions from the 34-identifier inventory from exclusion of the single short CSV; Table S2 preserves the complete mapping.

Acquisition-time PICO OS, ADB client, browser engine, and WebXR runtime versions were not retained. The archived platform package reports version 2.3, embedded Python 3.12.8, and websockets 16.0. These describe the retained package, not the acquisition-time installation. Table S1 separately records the environment used for this reanalysis. Session assignments follow the dated reports and export inventory in Table S2.

### 2.2. Documented Acquisition Implementations and Export Semantics

The acquisition archive described two implementations. An ADB reader parsed the most recent compatible acceleration, gyroscope, and quaternion records from Android sensorservice output and sent JSON frames to a local WebSocket endpoint. A browser collector supported WebXR or DeviceMotion. In the documented WebXR branch, translational velocity calculated from pose-position differences was placed in compatibility fields named gyro_x, gyro_y, and gyro_z; those names therefore do not ensure angular velocity. The DeviceMotion branch converted rotationRate from degrees per second to radians per second. The interface specifications describe these APIs [17,19,20,21], but do not verify which branch produced any particular CSV. The original acquisition modules are not included in the public derived-data snapshot.

The mapper calculated acceleration magnitude, gyro-field magnitude, and ZYX quaternion-to-Euler angles. Its acceleration excursion was *a_e_* = abs(accMag − 9.80665), using the mapper’s nominal acceleration scale. This scalar operation is not directional gravity separation. The CSV schema retained timestamp, accMag, gyroMag, roll, pitch, yaw, behavior, and confidence, but omitted source identity, raw axes, sensor-event timestamps, and quaternion. The revised analysis reader accesses only the six numeric fields; stored behavior and confidence are not inputs to the recalculated labels.

Timing groups were assigned after export. A median positive interval of no more than 0.12 s identified S001 and S009; the other 27 files had median intervals of 0.265–0.274 s. The labels browser-compatible and ADB-like indicate timing compatibility with documented implementations. They do not recover the historical source, distinguish WebXR from DeviceMotion, or establish the physical equivalence of gyroMag. Figure 1 summarizes the reanalysis of the retained exports and annotation records.

### 2.3. Timestamp and Exported-Field Quality Audit

For each session, positive export intervals were computed as Δ*t_i_* = *t_i_* − *t_i_*_−1_, and effective export rate as 1/median(Δ*t_i_*). The audit recorded zero or negative intervals, interval IQR, interval coefficient of variation, and maximum interval. A long interval was defined as Δ*t_i_* > max(1.0 s, 5 × median positive Δ*t*). Time-based windows reset after such an interval; no samples were inserted across it. The sum of long-interval spans is not a hardware-event-loss estimate, and the summed first-to-last span is not uninterrupted observation time.

We checked all six required numeric columns in all 30 CSVs for missing or non-finite values. Adjacent rows were compared across accMag, gyroMag, roll, pitch, and yaw at exported precision. Identical five-field pairs were retained because rounding, stationary observations, constant fields, and repeated upstream values cannot be distinguished without sensor-event timestamps. Their denominator is the number of within-session adjacent pairs.

A field was flagged as session-wide constant when its full-session range was no greater than 10^−6^. The term frozen in the evidence files denotes this numerical screen, not a confirmed hardware failure. The Euler-field set was flagged when all three angle ranges met the criterion. An accMag flag disabled the acceleration excursion rule; an Euler flag disabled smooth-turn and Euler-conditioned low-motion. Passing this screen does not establish calibration, correct units, or absence of intermittent stalls. All retained gyroMag series varied; the released rule implementation applies explicit acceleration and Euler gates, not an additional operative gyro gate for these files.

We distinguish two denominators. Whole-output composition uses all retained samples in a group, including samples for which a channel-dependent rule is disabled. Channel-eligible summaries exclude sessions flagged for the relevant channel. A disabled rule can therefore have a structural zero in whole-output composition without representing an observed absence of movement. No acceleration values are synthesized during reanalysis. Whether an optional fallback in the acquisition implementation was active cannot be recovered from the exported schema.

### 2.4. Time-Based Windows and Deterministic Outputs

The primary configuration includes samples in [*t* − 2.0 *s*, *t*] within the current uninterrupted segment. The available history is shorter at a session start or immediately after a reset. The 2.0 s duration is an engineering reference, not an annotation-optimized parameter; it contains approximately 7–8 observations at the dominant export rate. The window summaries are the population standard deviation of gyroMag and the largest arithmetic max-minus-min range among roll, pitch, and yaw. A shortest-containing-arc circular range is examined separately because arithmetic ranges can be inflated near ±180°.

Table S5 gives the complete conditions and priority order. At each sample, the first matching-enabled rule is assigned: rapid-turn, smooth-turn, moderate-turn, acceleration excursion, Euler-conditioned low-motion, then residual output. Rapid-turn uses current gyroMag > 1.5 or window SD > 0.8; smooth-turn additionally requires eligible Euler fields, 0.15 ≤ gyroMag ≤ 1.5, SD ≤ 0.6, and Euler range ≥ 3°. Moderate turn uses 0.3 ≤ gyroMag ≤ 1.5. Acceleration excursion requires eligible accMag and *a_e_* > 0.3. Euler-conditioned low-motion requires eligible Euler fields, gyroMag < 0.15, and Euler range < 2°. Thresholds are inherited numerical settings on exported fields; their pilot or literature-calibration record was not retained. Residual output is a heterogeneous fallback, not true inactivity.

A separate three-group gyro-based output is used for the annotation comparison. The rapid condition is evaluated first. Otherwise, gyroMag ≥ 0.15 gives ordinary head motion, and the remaining samples give low head motion. This is not a simple merging of the six-rule output labels: it omits Euler and acceleration requirements and assigns mutually exclusive groups using rapid-first precedence.

### 2.5. Configuration Comparisons, Denominators, and Statistics

Time-window durations of 0.5, 1.0, 2.0, and 4.0 s were compared with the same long-gap reset and channel gates. The no-window baseline uses the current sample alone, giving a population SD and eligible Euler range of zero. The archived sample-history branch includes indices max(0, *i* − 20) through i and does not reset after long gaps. We retained that branch as a historical configuration comparator and added a matched 21-sample branch, whose start is max(segment_start, *i* − 20). The matched branch uses the same full-session channel gates and long-gap threshold as the 2.0 s configuration. It separates sample-history duration from reset handling without changing thresholds or rule priority.

Two priority alternatives move moderate-turn before smooth-turn, or low-motion before acceleration excursion. Thresholds were scaled jointly from 0.7 to 1.3 in 0.1 steps; all 11 numerical boundaries were also perturbed individually by ±10%, ±20%, and ±30%. Circular versus arithmetic Euler range provides another implementation comparison. Each reported change is the number of samples with a different final rule output divided by the number of retained samples compared, using the 2.0 s configuration as reference. Conflict fractions count samples satisfying more than one enabled condition before priority resolution.

For session summaries, medians, IQRs, and ranges are reported. Median intervals use 5000 whole-session bootstrap resamples and percentile limits, with linear interpolation between ordered estimates; the median-summary random seed is 4505577 plus the number of sessions. No precision interval is reported for the two browser-compatible files, which are also shown individually. Sample-count-weighted output fractions use pooled sample counts within a timing group and are not elapsed-time fractions. Session-level fractions give equal weight to sessions. Neither statistic estimates a causal acquisition-path effect. Each session belongs to a different participant, so participant and session clustering coincide.

### 2.6. Video Annotation, Bin Construction, and Agreement

The saved alignment log contains 19 candidate third-person videos. Its selection rule requires correlation *r* ≥ 0.45 and a best-versus-next-peak margin ≥ 0.08. The historical script extracts frame-difference energy at 5 frames/s from 64 × 36 video, averages it by second, and compares it with one-second mean gyroMag. Empty gyro seconds are zero-filled. Integer-second offsets are searched while keeping the video within the CSV span; when the video is longer, offsets permitting partial overlap are allowed. At least 20 overlapping seconds are required. Correlation is computed from standardized overlapping sequences; the next peak must be more than 5 s from the best peak. Decisions use unrounded values, while the log displays three decimals. We reconstructed selection from that log and inspected the script, rather than rerunning video processing. S019 failed the correlation criterion; S023ΔS025, S027–S029, S031, and S034 failed the margin criterion. Table S8 lists all candidates. Selection may favor recordings with clearer motion correspondence.

The ten selected sessions were S015, S017, S018, S020, S021, S022, S026, S030, S032, and S033. All are ADB-like; all have flagged Euler fields and four have flagged accMag. Two project team raters returned separate forms under written instructions to annotate independently. Each rater completed one labeling pass, taking approximately two hours according to the study team’s retrospective account. The tool was revised once to align video with an IMU magnitude display. A separate training duration was not recorded. The first-pass interface used REVIEW = false to hide machine and prior-label columns and hid the alignment panel after confirmation. Return footers record blind_mode = 1, saw_machine = 0, and independent = 1. These are interface and self-report records; machine values remained accessible in page source, so the procedure is described as interface-masked rather than fully monitored blinding.

The analysis joins non-empty rater records on the exact session identifier and CSV-time start and end. Within each one-second half-open interval [start, end), the machine output is the most frequent sample label. Ties use rapid > ordinary > low for three groups and the six-rule priority order for six outputs. Intervals without machine samples are omitted. Human gaze and idle map to low head motion, head_turn to ordinary, and sudden_turn to rapid; these are the labels present in the paired records. Table S5 gives the exploratory six-output mapping. The 661 paired bins contain 2400 exported samples, or 5.27% of the retained 45,560 samples. Automatic overlap in seconds and paired-bin support are reported separately in Table S8.

For S021, the saved automatic offset is 65 s. Both original return files record video_anchor_seconds = −68.00, manual nudge operations, and alignment confirmation. Under the interface convention CSV time = video time − anchor, the saved final offset is therefore 68 s. This evidence establishes a recorded adjustment, not its accuracy or that each rater independently chose the offset. The earliest evaluated bin also starts at 68 s. As a sensitivity check, we shift only the S021 machine lookup back by 3 s while retaining the human labels; this does not constitute video realignment or new annotation.

We recomputed percentage agreement, Cohen’s kappa, and 95% intervals from the original paired returns. Intervals use 5000 resamples of the ten whole-session clusters, seed 4505577, retaining all bins and the multiplicity of each drawn session. Percentile limits summarize valid estimates. Leave-one-session-out analyses examine dependence on individual sessions. Complete three-group matrices and exploratory six-output matrices are supplied in Tables S11 and S12. These estimates describe the selected ADB-like subset, not an independent classifier test set.

### 2.7. Reanalysis and Reproducibility

The public repository preserves the current derived-data and analysis snapshot at commit 23c30dc17887a19fb4de14d9d87c4b3330ba60df [35]. This public snapshot contains the 8 September revision’s analysis scripts, recomputed evidence, figure sources, tests, and SHA-256 manifest. It records the original inputs required for a full rebuild; original motion CSVs, annotation returns, consent forms, identifiable video, and acquisition-platform source are held separately.

We reran export QC, time-window and threshold alternatives, angular-range checks, annotation pairing, and session-cluster bootstrap intervals from the original files. Additional analyses recover saved alignment metadata, quantify sample coverage and residual composition, and compare the gap-matched 21-sample history. Exact output counts and channel-eligible denominators are generated directly, not inferred from rounded fractions. The document builder requires passing evidence checks. Derived tables can be checked without participant originals; full source-level reanalysis requires the controlled inputs. The current public snapshot was released on 9 September 2026; no reuse license is granted.

## 3. Results

### 3.1. Record Flow and Export Timing

The retained files contain 45,560 samples over a summed first-to-last span of 11,605.8 s. Recomputed QC found no duplicate or nonmonotonic timestamps and identified 242 long intervals in 19 sessions, with 467.3 s of combined inter-arrival span. There were 988 identical five-field pairs among 45,531 within-session adjacent pairs (2.17%). These describe exported values and timing, not hardware-event loss or duplication. File-level results are in Table S3.

The 27 ADB-like sessions have a median export rate of 3.745 Hz (IQR 3.745–3.759; range 3.650–3.774). S001 and S009 have rates of 8.333 and 20.000 Hz and spans of 362.446 and 122.000 s, respectively. Table 3 presents those two observations separately rather than attaching a population precision interval to n = 2.

### 3.2. Constant Fields, Eligible Denominators, and Output Composition

Five ADB-like files—S015 and S017–S020—contain 5482 samples with accMag fixed at 9.7335 on the exported scale. Their gyroMag series vary, with full-session ranges of 2.8797–6.3172 and 284–738 distinct four-decimal values. The finding concerns the exported accMag field; it does not locate a fault in the sensor, service, reader, or transport. Twenty-eight sessions have constant roll, pitch, and yaw. Only S001 has exported Euler variation that passes this screen.

The acceleration-eligible subset contains 24 sessions and 40,078 samples overall, including 22 ADB-like sessions and 34,637 samples. Whole-output composition assigns 525 samples to acceleration excursion: 1.152% of all 45,560 samples, versus 1.310% of the 40,078 acceleration-eligible samples. In ADB-like files, the corresponding count is 441: 1.099% of all 40,119 samples or 1.273% of the 34,637 eligible samples. These are the final rule assignments after priority resolution, not all the occasions of when the acceleration condition was satisfied. Table S4A states both denominators.

Figure 2 shows whole-output composition for ADB-like files and for S001 and S009 individually.

All 1002 smooth-turn and 92 low-motion assignments come from S001; these rules are disabled in the other 28 retained sessions. Residual output accounts for 32,660 samples (71.69%), with a session median of 72.7% (IQR 61.7–79.7%). Within the residual category, 25,636 samples (78.49%) have unavailable Euler information and gyroMag < 0.15; 5172 (15.84%) have unavailable Euler information and 0.15 ≤ gyroMag < 0.30. A further 1850 (5.66%) fail the low-motion angle-range condition in S001, and two fail its smooth-turn conditions below the moderate threshold. This composition explains why the fallback cannot be interpreted as inactivity. Tables S4 and S4B retain the output and eligibility denominators.

### 3.3. Dependence on Window, Threshold, and Priority Choices

Table 4 summarizes configuration dependence. The no-window baseline changes 1.82% of ADB-like outputs and 52.66% in the two higher-rate files. With the same gap resets as the primary method, the 21-sample history changes 974/40,119 ADB-like outputs (2.428%) and 47/5441 higher-rate outputs (0.864%). The historical no-reset branch changes 1029 ADB-like outputs (2.565%). Adding resets to that branch changes 59 assignments across nine ADB-like sessions; no assignment changes in S001 or S009. Thus, the matched contrast measures history construction with gap handling held constant. The 0.5, 1.0, and 4.0 s alternatives vary duration within the same time-window implementation.

Moving moderate-turn ahead of smooth-turn changes 10.15% of the higher-rate files’ outputs and none in the Euler-flagged ADB-like group. Moving low-motion ahead of acceleration excursion changes no outputs. Before priority resolution, 3073 of 45,560 retained samples (6.74%) satisfy multiple enabled conditions. These file-level differences do not demonstrate a browser-path effect: there are only two higher-rate files, no paired acquisition, and no retained source identity.

Joint threshold scaling changes 3.49% of all outputs at −10%, 12.59% at −30%, 2.89% at +10%, and 7.50% at +30%. The largest one-at-a-time change is 6.3% when the moderate-turn lower boundary is reduced by 30%; the median session change is 6.8% (IQR 5.4–8.4%). Arithmetic and circular Euler ranges differ in 209 S001 windows, with a maximum reduction of 355.22°, but no final output changes. Zero sensitivity can reflect a disabled or non-winning condition, rather than a robust behavioral boundary. Tables S6 and S7A give the stratified configuration results; Table S13 describes adjacent-output persistence without crossing long-gap boundaries.

### 3.4. Agreement in the Selected Annotation Subset

The raters agree on 569 of 661 three-group bins (86.1%). Their kappa is 0.698, with a recomputed session-cluster interval of 0.615–0.777. The separate gyro-based output agrees with rater A on 511 bins (77.3%) and rater B on 514 bins (77.8%), giving kappas of 0.530 and 0.529 (Table 5). The complete count matrices are in Table S11; all three point estimates also match direct matrix calculations.

Shifting only the S021 machine lookup by −3 s leaves 660 bins because one interval contains no exported sample. Kappa becomes 0.530126 for rater A and 0.528759 for rater B, compared with 0.530413 and 0.529033 in the primary pairing. The rounded values are similar, but the comparison also changes the evaluated bin set. Leave-one-session-out kappas span 0.449–0.583 and 0.472–0.571. In the exploratory six-output mapping, rule-versus-rater kappa is 0.154 and 0.139. The three-group result concerns a separately defined output and should not be interpreted as improved performance of the six-rule system.

## 4. Discussion

### 4.1. What the Audit Establishes

For RQ1, the common CSV schema preserves sufficient information for an export-level timing and constant-field audit, but not enough to identify historical source branches or establish a common physical interpretation of every field. Keeping timing groups separate avoids treating a shared column name as a calibration record. The distinction between whole-output and channel-eligible denominators further prevents disabled rules from being interpreted as negative observations of behavior.

For RQ2, 21 samples span 20 intervals: approximately 5.34 s at the dominant export rate, 2.40 s in S001, and 1.00 s in S009. A timestamp-based window places the same maximum duration on each file. The matched 21-sample comparison holds gap handling and channel eligibility constant. Its 2.428% ADB-like change fraction differs from the historical branch’s 2.565%, showing why reset handling should be specified alongside window length. The additional reset-only comparison localizes the effect to 59 assignments rather than attributing the whole contrast to duration.

For RQ3, implementation choices affect software outputs, but their effects depend on available fields and priority competition. In particular, Euler-dependent outputs are unreachable in ADB-like files. A small change fraction or an unchanged label after circular-range correction therefore cannot validate a threshold or prove algorithmic robustness. The individual S001 and S009 profiles also show why two-file weighted summaries are insufficient for a population-level acquisition-method comparison.

For RQ4, the matrices support the reported agreement point estimates in ten selected ADB-like sessions. They do not supply ground truth for every six-rule label, cover the higher-rate files, or establish out-of-sample predictive performance. The narrower three-group taxonomy is useful for describing what the video comparison can observe, provided it remains explicitly separate from the six-rule vocabulary.

### 4.2. Relation to Existing Workflows

Supervised activity-recognition studies evaluate predictions against labeled data [5,7,11]. Our audit addresses the preceding question of whether an exported headset file retains usable field semantics, timing, and channel information. The shared failure mode is concrete: an absent orientation channel makes some rule outputs unreachable, and their apparent zeros depend on the denominator. UXF, OpenSense, and Lab Streaming Layer address complementary experiment, calibration, and synchronization needs (Table 1) [14,15,16]. API specifications describe interfaces but cannot recover the source of a historical file [17,18,19,20,21].

Optical motion capture adds a calibrated spatial reference, frame alignment, and measurable tracking error, with camera, space, and setup requirements [24,33,34]. The present files provide no equivalent reference. Their practical contribution is an inspectable set of export-level checks and examples of how missing metadata and channel gates alter downstream rule interpretation. Releasing derived evidence improves inspectability, while the continuing restriction of original records limits independent source-level verification.

### 4.3. Limitations and Further Validation

The timing labels are retrospective and source identity is missing. No synchronized same-session recording through both implementations is available. Acquisition-time versions of the operating system, ADB client, browser engine, and WebXR runtime were not retained (Table S1); builds are unknown; mapped magnitudes cannot reconstruct raw-axis calibration, directional gravity separation, sensor fusion, or hardware-event timing. The 27/2 imbalance and non-equivalent field semantics preclude an inferential acquisition-method comparison.

The 60 s completeness criterion was introduced retrospectively and was not preregistered. It separates the 8.410 s record from retained files of at least 122 s, but is not a general validity threshold. Each retained session represents a different participant. Nevertheless, the small selected annotation subset and the two higher-rate files limit generalization beyond this dataset.

The constant-field screen does not diagnose hardware failure or detect all intermittent stalls. Inherited thresholds lack a documented calibration basis, and sensitivity analysis does not supply that calibration. Although the added 21-sample comparison now controls gap resets, neither a matched configuration nor stable outputs establish behavioral validity. A constant scalar magnitude does not imply that every raw acceleration axis was constant.

Annotation is limited to ten participants selected for video availability and alignment correspondence. Saved candidate decisions and return-file offsets make selection traceable, but do not provide external timing validation. Third-person video cannot resolve every six-rule state. Labeling effort is author-reported; formal training duration and monitored-independence records remain unavailable. The S021 adjustment is documented, yet its lookup sensitivity is not a substitute for reference synchronization or new independent labels.

Further validation should retain source labels, raw axes, hardware timestamps, calibration and runtime records, and participant-to-session links; record both interfaces synchronously; and compare appropriate measurements with a calibrated reference. A classifier-performance study would additionally require observable labels and a participant-aware held-out design. These are separate validation tasks, not properties established by the current software audit.

## 5. Conclusions

The audit retained 29 sessions and 45,560 samples from 30 available exports in a study of 34 participants. It identified two timing profiles, five constant accMag series, and 28 constant Euler-field sets. Explicit channel eligibility separates missing rule inputs from observed output composition. Matched window comparisons isolate history construction from gap resets, while reconstructed annotation records support a bounded agreement analysis. Together, these steps provide a reproducible export-level audit without establishing sensor accuracy or validated behavioral classification.

## Figures and Tables

**Figure 1:**
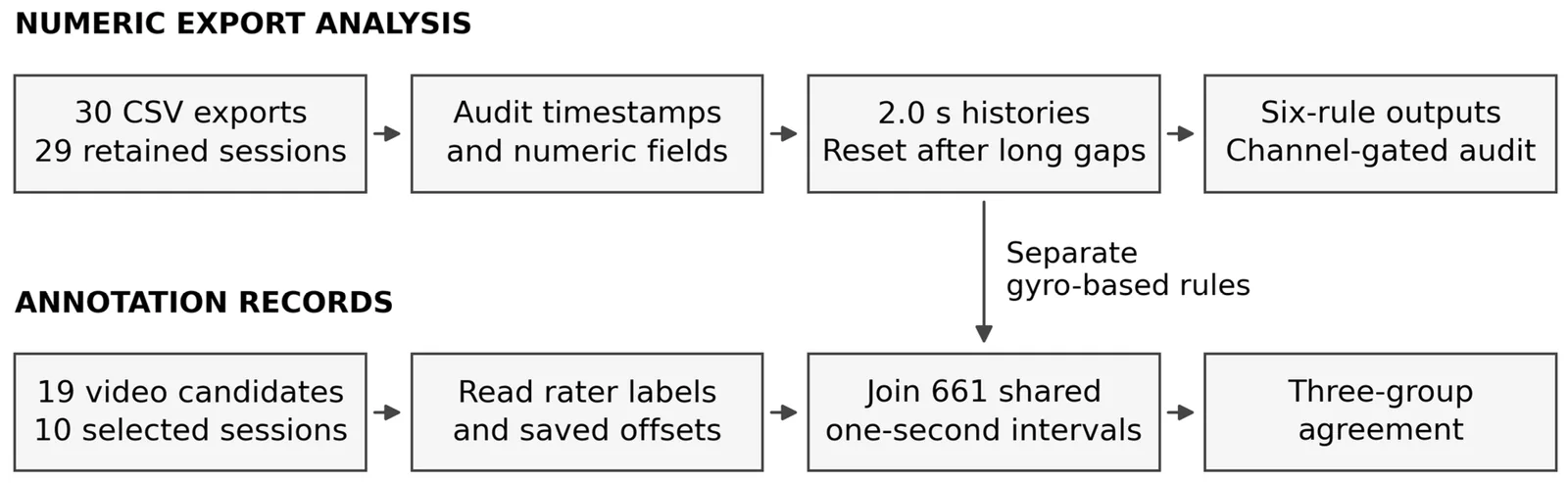
Reanalysis workflow. Exported numeric fields enter the timing audit and time-window analysis; stored behavior and confidence are excluded. The six-rule analysis applies channel gates, whereas annotation agreement uses a separate gyro-based three-group output. Saved alignment records identify the selected annotation subset, not independently validated synchronization.

**Figure 2:**
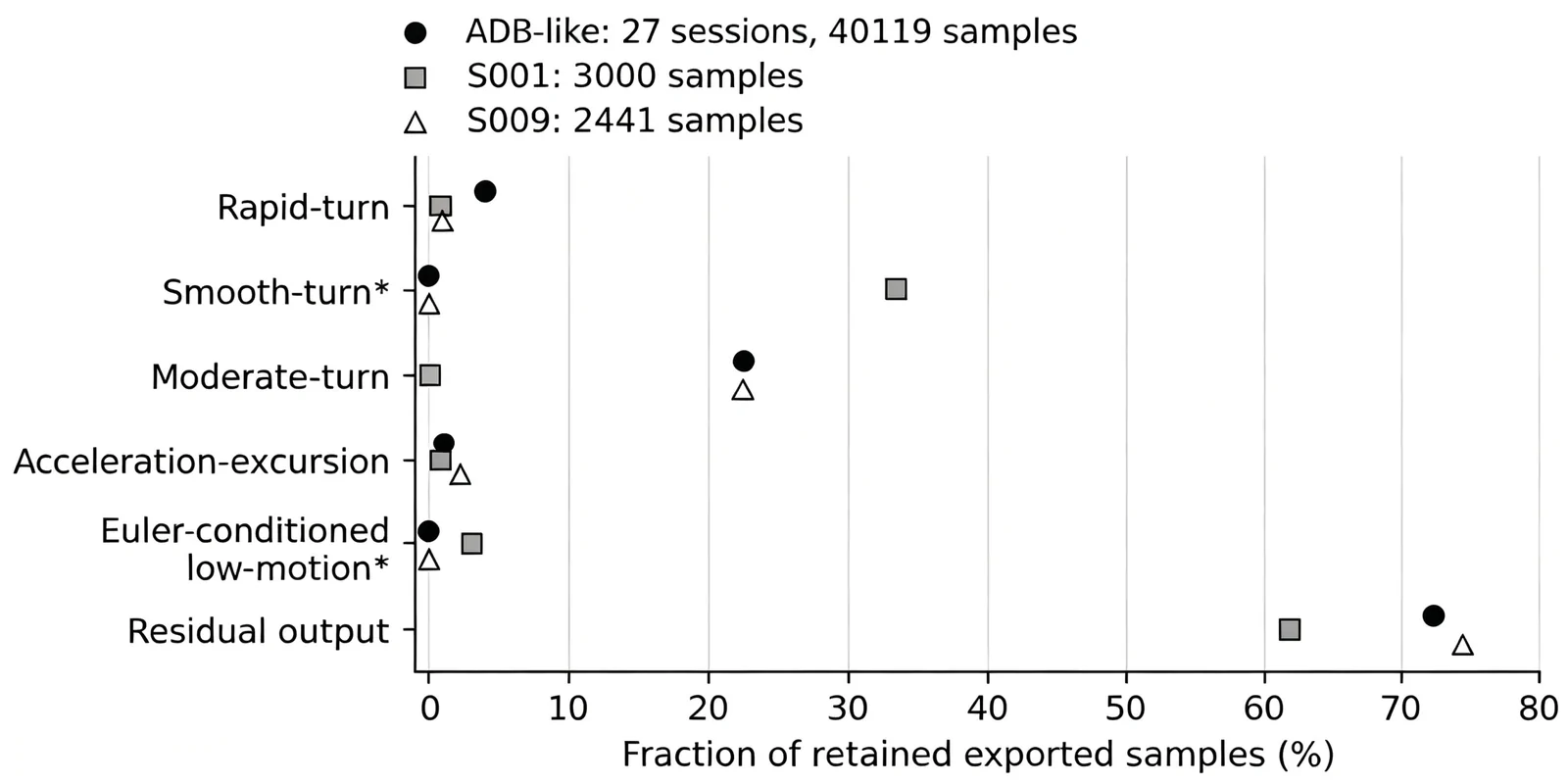
Whole-output composition using the complete retained-sample denominator for each series. The two browser-compatible sessions are shown separately. * Smooth-turn and Euler-conditioned low-motion are disabled in all ADB-like sessions and in S009; their zeros are structural. Acceleration-eligible denominators are reported separately in Table S4A. Residual output is not inactivity.

**Table 2:** Study-record flow. Counts of records, files, sessions, and samples are distinct.

Stage	Count	Definition or Identifiers
Participants providing consent	34	34 distinct participants; one participation each
Analysis CSV exports	30	Includes the short S002 record
Dated report files	36	20 HTML and 16 PDF; repeated reports for S001/S002
Identifiers without analysis CSV	4	S003, S004, S012, S016
CSV excluded by completeness criterion	1	S002: 842 samples, 8.410 s
Retained sessions	29	S001, S005–S011, S013–S015, S017–S034
Retained exported samples	45,560	Repeated observations within the retained sessions

**Table 3:** Export timing and constant-field flags. ADB-like values are session median (IQR); S001 and S009 are individual observations.

Group or Session	Sessions/Samples	Export Rate (Hz)	First-to-Last Span (s)	Maximum Gap (s) †	Flagged AccMag; Euler
ADB-like	27/40,119	3.745 (3.745–3.759)	439.9 (326.2–500.1)	1.955	5; 27
Browser-compatible: S001	1/3000	8.333	362.446	0.148	0; 0
Browser-compatible: S009	1/2441	20.000	122.000	0.050	0; 1

† The ADB-like entry is the median of per-session maximum gaps. Flags indicate session-wide constant exported values, not confirmed hardware failure. No precision interval is assigned to the two higher-rate files.

**Table 4:** Changed final outputs relative to the primary 2.0 s configuration.

Configuration	ADB-Like (%)	Higher-Rate Files (%)	Session Median (%) [IQR]
Current-sample/no-window	1.82	52.66	1.55 [0.56–3.27]
21 samples; matched gap reset	2.43	0.86	1.41 [0.74–3.95]
21 samples; historical no reset	2.56	0.86	1.57 [0.74–4.24]
0.5 s window	1.75	11.78	1.38 [0.55–3.05]
1.0 s window	1.44	4.50	1.18 [0.42–2.53]
4.0 s window	1.77	2.13	1.13 [0.69–3.17]
Moderate before smooth	0.00	10.15	0.00 [0.00–0.00]
Low before excursion	0.00	0.00	0.00 [0.00–0.00]
Circular Euler range	0.00	0.00	0.00 [0.00–0.00]

Group percentages use 40,119 and 5441 samples; session summaries use all 29 retained sessions. Both 21-sample branches use the same full-session channel gates as the primary method. The matched branch resets after long gaps; the historical branch does not. Differences are software-output changes, not accuracy estimates.

**Table 5:** Agreement of paired human labels and the separate three-group gyro-based output.

Comparison	Matching/Paired Bins	Agreement (%)	Kappa	Session-Cluster 95% CI
Rater A versus rater B	569/661	86.1	0.698	0.615–0.777
Three-group output versus rater A	511/661	77.3	0.530	0.275–0.657
Three-group output versus rater B	514/661	77.8	0.529	0.317–0.618

All 10 sessions are ADB-like, with flagged Euler fields; four also have flagged accMag. These comparisons use the separate three-group output. Intervals were recomputed with 5000 whole-session resamples. Each session corresponds to a different participant, so session and participant clusters coincide in this dataset.

## Data Availability

The current public derived-data snapshot is available at commit 23c30dc17887a19fb4de14d9d87c4b3330ba60df [35]. The repository provides the current scripts, derived evidence, figures, and input/output hashes. Original data supporting the findings are available from the corresponding author upon reasonable request, subject to participant consent, privacy protection, and institutional ethics requirements. Identifiable records are not bundled. No reuse license is granted; code, data, and figure permissions remain reserved by the authors.

## Supplementary Materials

The following supporting information can be downloaded at: https://www.mdpi.com/article/10.3390/s26196151/s1, Tables S1–S13, including S4A, S4B, and S7A, provide software-version provenance, participant/session flow, per-session QC, eligible denominators, rule definitions, recomputed configuration results, all alignment candidates, and agreement matrices. Complete numerical evidence, the residual breakdown, and aggregate transition counts are also supplied as machine-readable files in the revision package.

## Abbreviations

ADB, Android Debug Bridge; CSV, Comma-Separated Values; HMD, Head-Mounted Display; IMU, Inertial Measurement Unit; VR, Virtual Reality; WebXR, Web Extended Reality.
