# Supplementary material for: Architecture and Behavior-State Classification of a WebSocket-Based IMU Stream Extraction Workflow for Pico 4 Headsets
Source: Sensors (Basel). 2026 Sep 28;26(19):6151. doi: 10.3390/s26196151 (PMC13667537; doi:10.3390/s26196151)
Supplement: Supplementary file 1 [file sensors-26-06151-s001.zip › sensors-4505577-supplementary.pdf]

Supplementary Materials

A Quality Audit of Pico 4 Pro Motion Exports and Time-Based Rule Outputs

Scientific revision: 8 September 2026. Editorial update: 21 September 2026 (software identification and supplementary-table numbering). QC, rule alternatives, annotation pairing, and bootstrap intervals were recomputed for the scientific revision from controlled original files; numerical results are unchanged in this editorial update.

Exact counts and channel eligibility are calculated directly from the reanalysis. The added analyses recover all candidate-selection records, saved return-file offsets, sample coverage, and a reset-matched 21-sample comparator. Historical video cross-correlation was inspected through its saved log and script, not rerun. All two-file results remain descriptive.

Table S1. Software-version provenance.

Scope	Component	Value	Evidence boundary
Acquisition	Headset model	Pico 4 Pro	Author-reported device identity; not a metrological result
Acquisition	PICO OS build	Not retained	No acquisition-time record supplied
Acquisition	ADB client / platform-tools	Not retained	No acquisition-time version output supplied
Acquisition	Browser engine / WebXR runtime	Not retained	No acquisition-time engine/runtime record supplied
Revision archive	Platform code	2.3	Recorded from project.config.json in the original revision; acquisition modules are not in the public snapshot
Revision archive	Bundled Python	3.12.8	Recorded in the original revision archive, not evidence of acquisition-time software
Revision archive	websockets	16.0	Recorded in the original revision archive, not evidence of acquisition-time software
Public derivative snapshot	Repository revision	23c30dc17887a19fb4de14d9d87c4b333 0ba60df	8 September scientific revision; public synchronization on 9 September 2026
This reanalysis	Python / python-docx / matplotlib	3.12.14 / 1.2.0 / 3.10.6	Executed revision environment; not acquisition-time software

Table S2. Complete study-record and session flow.

ID	Consent record*	CSV present in inventory	Export ID	Report files	Retained	Exclusion reason
S001	Yes	Yes	1776821554249	5	Yes	
S002	Yes	Yes	1776754772978	2	No	CSV export incomplete (8.410 s)
S003	Yes	No		0	No	No analysis CSV export retained
S004	Yes	No		0	No	No analysis CSV export retained
S005	Yes	Yes	1776824662730	1	Yes	
S006	Yes	Yes	1776825800472	1	Yes	

ID	Consent record*	CSV present in inventory	Export ID	Report files	Retained	Exclusion reason
S007	Yes	Yes	1776827240332	1	Yes	
S008	Yes	Yes	1776828552202	1	Yes	
S009	Yes	Yes	1776830107430	1	Yes	
S010	Yes	Yes	1776842825545	1	Yes	
S011	Yes	Yes	1776844051112	1	Yes	
S012	Yes	No		1	No	Dated PDF report retained, but no corresponding analysis CSV
S013	Yes	Yes	1776846533989	1	Yes	
S014	Yes	Yes	1776847632327	1	Yes	
S015	Yes	Yes	1776991265337	1	Yes	
S016	Yes	No		0	No	No analysis CSV export retained
S017	Yes	Yes	1776998386020	1	Yes	
S018	Yes	Yes	1776999562464	1	Yes	
S019	Yes	Yes	1777000526552	1	Yes	
S020	Yes	Yes	1777002144701	1	Yes	
S021	Yes	Yes	1777010515392	1	Yes	
S022	Yes	Yes	1777011363810	1	Yes	
S023	Yes	Yes	1777012565224	1	Yes	
S024	Yes	Yes	1777013387851	1	Yes	
S025	Yes	Yes	1777014295491	1	Yes	
S026	Yes	Yes	1777017440813	1	Yes	
S027	Yes	Yes	1777019914575	1	Yes	
S028	Yes	Yes	1777022706323	1	Yes	
S029	Yes	Yes	1777023729187	1	Yes	
S030	Yes	Yes	1777024675993	1	Yes	
S031	Yes	Yes	1777025679136	1	Yes	
S032	Yes	Yes	1777026812944	1	Yes	

ID	Consent record*	CSV present in inventory	Export ID	Report files	Retained	Exclusion reason
S033	Yes	Yes	1777027785844	1	Yes	
S034	Yes	Yes	1777028938686	1	Yes	

* The author confirms 34 distinct participants, each taking part once, with signed consent. Five identifiers are excluded from analysis; only one of the 30 actual CSV exports is excluded. S012 has a report without an analysis CSV. Each of the 29 retained sessions corresponds to a different participant.

Table S3. Per-session export-quality audit.

ID	Retained	Samples	Span (s)	Rate (Hz)	Interval CV	Long gaps	Max gap (s)	Identical pairs	Timing group	accMag flagged	Euler flagged
S001	Yes	3,000	362.446	8.333	0.061	0	0.148	0	browser-compatible	No	No
S002	No	842	8.410	100.000	0.000	0	0.010	193	browser-compatible	No	No
S005	Yes	2,016	539.451	3.745	0.027	0	0.310	3	ADB-like	No	Yes
S006	Yes	1,879	500.924	3.745	0.025	0	0.320	0	ADB-like	No	Yes
S007	Yes	1,736	463.461	3.745	0.025	0	0.299	0	ADB-like	No	Yes
S008	Yes	1,860	497.167	3.745	0.025	0	0.320	0	ADB-like	No	Yes
S009	Yes	2,441	122.000	20.000	0.000	0	0.050	12	browser-compatible	No	Yes
S010	Yes	1,821	487.410	3.759	0.043	0	0.379	0	ADB-like	No	Yes
S011	Yes	1,638	439.913	3.745	0.048	0	0.385	1	ADB-like	No	Yes
S013	Yes	1,450	387.194	3.759	0.040	0	0.411	1	ADB-like	No	Yes
S014	Yes	1,831	489.983	3.759	0.044	0	0.382	1	ADB-like	No	Yes
S015	Yes	946	266.976	3.759	0.573	9	1.973	168	ADB-like	Yes	Yes
S017	Yes	1,159	327.605	3.731	0.547	10	2.022	69	ADB-like	Yes	Yes
S018	Yes	1,178	335.396	3.717	0.566	11	1.988	14	ADB-like	Yes	Yes
S019	Yes	1,136	328.189	3.650	0.554	10	2.018	46	ADB-like	Yes	Yes
S020	Yes	1,063	306.854	3.676	0.571	10	2.089	98	ADB-like	Yes	Yes
S021	Yes	1,163	327.034	3.745	0.547	10	1.947	43	ADB-like	No	Yes
S022	Yes	1,114	315.999	3.745	0.589	11	2.012	16	ADB-like	No	Yes
S023	Yes	1,134	319.411	3.745	0.556	10	1.997	12	ADB-like	No	Yes
S024	Yes	1,146	325.699	3.731	0.579	11	1.987	25	ADB-like	No	Yes
S025	Yes	1,156	326.689	3.745	0.580	11	1.991	13	ADB-like	No	Yes
S026	Yes	1,080	306.314	3.759	0.597	11	1.999	1	ADB-like	No	Yes

ID	Retained	Samples	Span (s)	Rate (Hz)	Interval CV	Long gaps	Max gap (s)	Identical pairs	Timing group	accMag flagged	Euler flagged
S027	Yes	1,837	515.620	3.774	0.561	17	1.949	153	ADB-like	No	Yes
S028	Yes	968	271.896	3.774	0.561	9	1.946	61	ADB-like	No	Yes
S029	Yes	1,814	509.836	3.774	0.564	17	1.934	15	ADB-like	No	Yes
S030	Yes	1,887	531.484	3.774	0.568	18	1.965	18	ADB-like	No	Yes
S031	Yes	1,808	509.508	3.759	0.569	17	1.955	26	ADB-like	No	Yes
S032	Yes	1,797	505.960	3.759	0.567	17	1.970	105	ADB-like	No	Yes
S033	Yes	1,773	499.362	3.774	0.569	17	1.976	10	ADB-like	No	Yes
S034	Yes	1,729	485.979	3.774	0.559	16	1.950	77	ADB-like	No	Yes

Span is last minus first export timestamp. Identical pairs compare the five exported motion fields, with samples minus one as the per-session denominator. Both flag columns use a full-session range $\leq 10^{-6}$; Euler requires all three fields to be constant. S002 is included in the file inventory but excluded from retained-session statistics. All values were recomputed from the original CSVs.

Table S4. Session summaries. Panel A: export timing.

Group	Metric	Sessions	Median (IQR)	Range	Median 95% CI
all	Duration, s	29	387.194 (325.699-499.362)	122.000-539.451	327.034-489.983
all	Export rate, Hz	29	3.759 (3.745-3.774)	3.650-20.000	3.745-3.759
all	Interval CV	29	0.559 (0.044-0.569)	0.000-0.597	0.061-0.567
all	Maximum gap, s	29	1.950 (0.382-1.988)	0.050-2.089	0.411-1.976
all	Long-gap span, s	29	19.425 (0.000-21.515)	0.000-34.384	0.000-21.512
ADB-like	Duration, s	27	439.913 (326.194-500.143)	266.976-539.451	327.034-497.167
ADB-like	Export rate, Hz	27	3.745 (3.745-3.759)	3.650-3.774	3.745-3.759
ADB-like	Interval CV	27	0.561 (0.046-0.569)	0.025-0.597	0.547-0.568
ADB-like	Maximum gap, s	27	1.955 (0.398-1.990)	0.299-2.089	1.934-1.987
ADB-like	Long-gap span, s	27	19.797 (0.000-25.996)	0.000-34.384	17.167-21.513

Panel A uses 5,000-resample session-bootstrap intervals. S001/S009 are shown individually in Table S3 and main Table 3; no two-file precision interval is reported.

Table S4 (continued). Panel B: composition of all final six-rule outputs.

Group	Final output	Sessions	Session median (IQR)	Range	Median 95% CI	Assigned / all samples	Sample fraction
all	Rapid turn	29	2.9% (1.1%-6.5%)	0.2%-14.6%	2.1%-5.1%	1,722 / 45,560	3.780%
all	Smooth turn	29	0.0% (0.0%-0.0%)	0.0%-33.4%	0.0%-0.0%	1,002 / 45,560	2.199%

Group	Final output	Sessions	Session median (IQR)	Range	Median 95% CI	Assigned / all samples	Sample fraction
all	Moderate turn	29	22.7% (18.0%-26.4%)	0.1%-41.4%	18.9%-25.2%	9,559 / 45,560	20.981%
all	Acceleration excursion	29	0.0% (0.0%-2.0%)	0.0%-5.6%	0.0%-0.9%	525 / 45,560	1.152%
all	Low motion	29	0.0% (0.0%-0.0%)	0.0%-3.1%	0.0%-0.0%	92 / 45,560	0.202%
all	Residual output	29	72.7% (61.7%-79.7%)	48.7%-92.0%	68.3%-74.8%	32,660 / 45,560	71.686%
ADB-like	Rapid turn	27	3.4% (2.0%-6.6%)	0.2%-14.6%	2.2%-6.1%	1,675 / 40,119	4.175%
ADB-like	Smooth turn	27	0.0% (0.0%-0.0%)	0.0%-0.0%	0.0%-0.0%	0 / 40,119	0.000%
ADB-like	Moderate turn	27	22.7% (18.3%-27.1%)	6.8%-41.4%	18.9%-26.3%	9,011 / 40,119	22.461%
ADB-like	Acceleration excursion	27	0.0% (0.0%-1.9%)	0.0%-5.6%	0.0%-0.0%	441 / 40,119	1.099%
ADB-like	Low motion	27	0.0% (0.0%-0.0%)	0.0%-0.0%	0.0%-0.0%	0 / 40,119	0.000%
ADB-like	Residual output	27	72.7% (62.0%-79.9%)	48.7%-92.0%	68.3%-76.6%	28,992 / 40,119	72.265%

Panel B uses exact counts from the recomputed outputs. Whole-output denominators retain sessions with disabled channel-dependent rules. Median intervals use 5,000 whole-session draws and seed 4505577 + n. Euler-dependent zeros in ADB-like files are structural, not observed absences of behavior.

Table S4A. Whole-output and channel-eligible denominators for channel-dependent rules.

Group	Output	All sessions / samples	Eligible sessions / samples	Final assignments	Fraction of all samples	Fraction of eligible samples
all	Smooth turn	29 / 45,560	1 / 3,000	1,002	2.199%	33.400%
all	Acceleration excursion	29 / 45,560	24 / 40,078	525	1.152%	1.310%
all	Low motion	29 / 45,560	1 / 3,000	92	0.202%	3.067%
ADB-like	Smooth turn	27 / 40,119	0 / 0	0	0.000%	-
ADB-like	Acceleration excursion	27 / 40,119	22 / 34,637	441	1.099%	1.273%
ADB-like	Low motion	27 / 40,119	0 / 0	0	0.000%	-
browser-compatible	Smooth turn	2 / 5,441	1 / 3,000	1,002	18.416%	33.400%
browser-compatible	Acceleration excursion	2 / 5,441	2 / 5,441	84	1.544%	1.544%
browser-compatible	Low motion	2 / 5,441	1 / 3,000	92	1.691%	3.067%

Eligibility is determined by the corresponding accMag or Euler screen. An eligible-sample fraction is not computed when no eligible sample exists (—). Counts are final assignments after priority resolution, not counts of all satisfied conditions. Fractions conditional on different channels do not form a common six-category composition.

Table S4B. Individual higher-rate files: final-output composition.

Output	S001 count	S001 / 3,000	S009 count	S009 / 2,441
Rapid turn	24	0.800%	23	0.942%
Smooth turn	1,002	33.400%	0	0.000%
Moderate turn	2	0.067%	546	22.368%
Acceleration excursion	28	0.933%	56	2.294%
Low motion	92	3.067%	0	0.000%
Residual output	1,852	61.733%	1,816	74.396%

S001 and S009 are timing-compatible labels, not verified browser acquisition routes. S009 has flagged Euler fields; smooth-turn and low-motion are disabled there. No bootstrap interval is attached to this two-file comparison.

Table S5. Complete deterministic rule sequence.

Priority	Canonical output	Condition	Implemented eligibility
1	rapid_turn_rule	gyroMag > 1.5 OR window SD > 0.8	No additional gyro gate in the released classifier; all retained gyroMag series vary
2	smooth_turn_rule	0.15 ≤ gyroMag ≤ 1.5; SD ≤ 0.6; Euler range ≥ 3°	Euler set not flagged
3	moderate_turn_rule	0.3 ≤ gyroMag ≤ 1.5	No additional gyro gate in the released classifier
4	acceleration_excursion_rule	abs(accMag − 9.80665) > 0.3	accMag not flagged
5	low_motion_rule	gyroMag < 0.15; Euler range < 2°	Euler set not flagged
6	residual_rule_output	No preceding enabled rule matches	Heterogeneous fallback

Assign the first enabled matching rule. SD is the population standard deviation; Euler range is the maximum of the three within-window arithmetic ranges. Thresholds operate on exported fields and are not calibrated behavioral boundaries. A separate three-group output evaluates rapid first, then ordinary if gyroMag ≥ 0.15, else low. This is not a merge of final six-rule outputs.

Human-label mapping: gaze → low_motion_rule; idle → residual_rule_output; head_turn → moderate_turn_rule; sudden_turn → rapid_turn_rule; scanning or scan/turn → smooth_turn_rule; postural_shift → acceleration_excursion_rule. The paired records use gaze, idle, head_turn, and sudden_turn. In the coarse comparison, gaze/idle → low, head_turn → ordinary, and sudden_turn → rapid.

Bins are [start, end) in CSV time. Majority-label ties use rapid > ordinary > low for three groups and the listed priority for six outputs. Rater rows are joined by exact session/start/end; empty machine bins are omitted. The no-window baseline has one sample (SD = 0; eligible Euler range = 0). Time-window and matched 21-sample variants reset after long gaps. The historical 21-sample branch does not. Full-session channel eligibility is identical across configurations.

Table S6. Recomputed configuration comparisons, including the reset-matched 21-sample history.

Configuration	Timing group	Sessions	Samples	Sample-weighted change	Median session change	Conflict fraction
Current-sample / no-window	all	29	45,560	7.89%	1.55%	4.94%
Current-sample / no-window	ADB-like	27	40,119	1.82%	1.55%	4.83%

Configuration	Timing group	Sessions	Samples	Sample-weighted change	Median session change	Conflict fraction
Current-sample / no-window	browser-compatible	2	5,441	52.66%	47.75%	5.75%
21 samples; matched gap reset	all	29	45,560	2.24%	1.41%	6.99%
21 samples; matched gap reset	ADB-like	27	40,119	2.43%	1.41%	6.03%
21 samples; matched gap reset	browser-compatible	2	5,441	0.86%	0.78%	14.02%
21 samples; historical no reset	all	29	45,560	2.36%	1.57%	7.01%
21 samples; historical no reset	ADB-like	27	40,119	2.56%	1.60%	6.06%
21 samples; historical no reset	browser-compatible	2	5,441	0.86%	0.78%	14.02%
0.5 s window	all	29	45,560	2.95%	1.38%	6.07%
0.5 s window	ADB-like	27	40,119	1.75%	1.38%	5.01%
0.5 s window	browser-compatible	2	5,441	11.78%	10.69%	13.84%
1.0 s window	all	29	45,560	1.80%	1.18%	6.38%
1.0 s window	ADB-like	27	40,119	1.44%	1.18%	5.34%
1.0 s window	browser-compatible	2	5,441	4.50%	4.08%	13.99%
2.0 s reference	all	29	45,560	0.00%	0.00%	6.74%
2.0 s reference	ADB-like	27	40,119	0.00%	0.00%	5.76%
2.0 s reference	browser-compatible	2	5,441	0.00%	0.00%	14.00%
4.0 s window	all	29	45,560	1.81%	1.13%	6.89%
4.0 s window	ADB-like	27	40,119	1.77%	1.13%	5.95%
4.0 s window	browser-compatible	2	5,441	2.13%	1.93%	13.84%
Moderate before smooth	all	29	45,560	1.21%	0.00%	6.74%
Moderate before smooth	ADB-like	27	40,119	0.00%	0.00%	5.76%
Moderate before smooth	browser-compatible	2	5,441	10.15%	9.20%	14.00%
Low before excursion	all	29	45,560	0.00%	0.00%	6.74%
Low before excursion	ADB-like	27	40,119	0.00%	0.00%	5.76%
Low before excursion	browser-compatible	2	5,441	0.00%	0.00%	14.00%
Circular Euler range	all	29	45,560	0.00%	0.00%	6.74%
Circular Euler range	ADB-like	27	40,119	0.00%	0.00%	5.76%
Circular Euler range	browser-compatible	2	5,441	0.00%	0.00%	14.00%

All alternatives were rerun from the original exports. The matched 21-sample branch shares the primary reset rule; the historical branch does not. Adding resets changes 59 historical-branch assignments, all in nine ADB-like sessions. Conflict is the fraction satisfying multiple enabled conditions before priority resolution. Two-file medians are descriptive; zero change can reflect a disabled or non-winning rule.

Table S7. Recomputed joint threshold-scaling sensitivity.

Joint change	Timing group	Sessions	Samples	Changed final outputs
-30%	all	29	45,560	12.59%
-30%	ADB-like	27	40,119	12.71%
-30%	browser-compatible	2	5,441	11.67%
-20%	all	29	45,560	7.79%
-20%	ADB-like	27	40,119	7.90%
-20%	browser-compatible	2	5,441	6.95%
-10%	all	29	45,560	3.49%
-10%	ADB-like	27	40,119	3.55%
-10%	browser-compatible	2	5,441	3.05%
0%	all	29	45,560	0.00%
0%	ADB-like	27	40,119	0.00%
0%	browser-compatible	2	5,441	0.00%
10%	all	29	45,560	2.89%
10%	ADB-like	27	40,119	2.92%
10%	browser-compatible	2	5,441	2.68%
20%	all	29	45,560	5.28%
20%	ADB-like	27	40,119	5.26%
20%	browser-compatible	2	5,441	5.46%
30%	all	29	45,560	7.50%
30%	ADB-like	27	40,119	7.45%
30%	browser-compatible	2	5,441	7.90%

The denominator is all retained samples in the indicated group; thresholds are scaled jointly. These are changes in deterministic output, not accuracy estimates. The nominal configuration is the 2.0 s, gap-reset, channel-gated reference.

Table S7A. Largest recomputed one-at-a-time effect for each boundary.

Timing group	Boundary	Nominal	Perturbation at largest effect	Sample-weighted change	Session median (IQR)
all	low gyro max	0.15	-30%	0.01%	0.00% (0.00%-0.00%)
all	low euler delta max	2	-30%	0.09%	0.00% (0.00%-0.00%)
all	smooth gyro min	0.15	-30%	0.48%	0.00% (0.00%-0.00%)
all	smooth gyro max	1.5	-30%	0.08%	0.00% (0.00%-0.00%)
all	smooth gyro std max	0.6	-30%	0.10%	0.00% (0.00%-0.00%)
all	smooth euler delta min	3	30%	0.01%	0.00% (0.00%-0.00%)
all	rapid gyro min	1.5	-30%	2.54%	2.56% (1.23%-3.89%)
all	rapid gyro std min	0.8	-30%	3.25%	2.65% (1.47%-4.95%)
all	moderate gyro min	0.3	-30%	6.26%	6.76% (5.38%-8.36%)
all	moderate gyro max	1.5	-30%	2.46%	2.56% (1.22%-3.89%)
all	acc excursion min	0.3	-30%	1.03%	0.00% (0.00%-1.64%)
ADB-like	low gyro max	0.15	-30%	0.00%	0.00% (0.00%-0.00%)
ADB-like	low euler delta max	2	-30%	0.00%	0.00% (0.00%-0.00%)
ADB-like	smooth gyro min	0.15	-30%	0.00%	0.00% (0.00%-0.00%)
ADB-like	smooth gyro max	1.5	-30%	0.00%	0.00% (0.00%-0.00%)
ADB-like	smooth gyro std max	0.6	-30%	0.00%	0.00% (0.00%-0.00%)
ADB-like	smooth euler delta min	3	-30%	0.00%	0.00% (0.00%-0.00%)
ADB-like	rapid gyro min	1.5	-30%	2.63%	2.64% (1.41%-3.94%)
ADB-like	rapid gyro std min	0.8	-30%	3.55%	2.68% (1.75%-5.13%)
ADB-like	moderate gyro min	0.3	-30%	6.72%	6.77% (5.52%-8.38%)
ADB-like	moderate gyro max	1.5	-30%	2.63%	2.64% (1.41%-3.94%)
ADB-like	acc excursion min	0.3	-30%	0.97%	0.00% (0.00%-1.49%)
browser-compatible	low gyro max	0.15	-30%	0.06%	0.05% (0.03%-0.07%)
browser-compatible	low euler delta max	2	-30%	0.79%	0.72% (0.36%-1.07%)
browser-compatible	smooth gyro min	0.15	-30%	3.99%	3.62% (1.81%-5.42%)
browser-compatible	smooth gyro max	1.5	-30%	0.68%	0.62% (0.31%-0.92%)
browser-compatible	smooth gyro std max	0.6	-30%	0.83%	0.75% (0.38%-1.12%)
browser-compatible	smooth euler delta min	3	30%	0.11%	0.10% (0.05%-0.15%)

Timing group	Boundary	Nominal	Perturbation at largest effect	Sample-weighted change	Session median (IQR)
browser-compatible	rapid gyro min	1.5	-30%	1.82%	1.89% (1.56%-2.21%)
browser-compatible	rapid gyro std min	0.8	-30%	1.01%	1.12% (0.58%-1.67%)
browser-compatible	moderate gyro min	0.3	-30%	2.89%	3.21% (1.64%-4.78%)
browser-compatible	moderate gyro max	1.5	-30%	1.14%	1.27% (0.64%-1.91%)
browser-compatible	acc excursion min	0.3	-30%	1.49%	1.56% (1.21%-1.91%)

All tested perturbations were ±10%, ±20%, and ±30%. Results were recomputed with the nominal 2.0 s, gap-reset, channel-gated reference. Full results are in threshold_one_at_a_time.csv. The two-file median/IQR is descriptive, not a population precision estimate.

Table S8. All alignment candidates, saved final offsets, and paired-bin support.

Session	Automatic offset (s)	Final offset A / B (s)	Correlation r	Peak margin	Overlap (s)	Paired bins	Decision
S015	196	196 / 196	0.801	0.081	53	50	Retained
S017	139	139 / 139	0.523	0.085	79	76	Retained
S018	123	123 / 123	0.588	0.343	137	131	Retained
S019	47	-	0.408	0.222	166	0	Excluded: r
S020	169	169 / 169	0.637	0.201	123	118	Retained
S021	65	68 / 68	0.594	0.121	64	63	Retained
S022	46	46 / 46	0.572	0.208	84	82	Retained
S023	162	-	0.680	0.068	59	0	Excluded: margin
S024	61	-	0.496	0.051	55	0	Excluded: margin
S025	84	-	0.490	0.004	66	0	Excluded: margin
S026	4	4 / 4	0.703	0.102	48	47	Retained
S027	0	-	0.755	0.024	42	0	Excluded: margin
S028	124	-	0.701	0.071	40	0	Excluded: margin
S029	154	-	0.633	0.012	31	0	Excluded: margin
S030	120	120 / 120	0.648	0.085	31	30	Retained
S031	131	-	0.817	0.026	34	0	Excluded: margin
S032	458	458 / 458	0.898	0.177	34	32	Retained
S033	78	78 / 78	0.703	0.091	33	32	Retained
S034	216	-	0.733	0.019	33	0	Excluded: margin

Automatic overlap is in seconds, not paired bins. The ten selected candidates have 686 s of automatic overlap and 661 paired bins covering 2,400 exported samples (5.27% of retained samples). Decisions require $r \geq 0.45$ and margin ≥ 0.08. S019 fails r; the other eight excluded candidates fail margin. Final offsets come from the original return footers, not the earliest non-empty bin. Both S021 returns contain a final 68 s offset, nudge operations, and confirmation, compared with the automatic 65 s candidate. This documents a saved adjustment, not independently verified timing accuracy or independent choice by each rater. Empty final-offset entries denote excluded candidates without paired returns in this analysis.

The first-pass interface records REVIEW=false and hidden machine/prior-label columns. All 20 paired return files record blind_mode=1, saw_machine=0, and independent=1; these are interface/export and self-report records, not monitored blinding. The author reports one labeling pass per rater, approximately two hours each, and one tool adjustment to align video with the IMU magnitude graph. No separate training duration or accuracy-improvement experiment is documented. See annotation_return_metadata.csv for anonymous metadata and source hashes.

Table S9. Prevalence in the separate three-group comparison.

Source	Class	Count	Fraction
annotator A	low head motion	446	67.5%
annotator A	ordinary head motion	171	25.9%
annotator A	rapid head motion	44	6.7%
annotator B	low head motion	462	69.9%
annotator B	ordinary head motion	162	24.5%
annotator B	rapid head motion	37	5.6%
coarse rule output	low head motion	428	64.8%
coarse rule output	ordinary head motion	194	29.3%
coarse rule output	rapid head motion	39	5.9%

Each source has 661 bins. Rounded percentages need not sum exactly to 100%. Human classes are recoded independently of the separate gyro-based output.

Table S10. Agreement sensitivity to a lookup shift and session omission.

Comparison	Scenario	Bins	Agreement	Kappa or range
rule output vs rater A	Primary saved offsets	661	77.3%	0.530413
rule output vs rater A	S021 lookup -3 s	660	77.3%	0.530126
rule output vs rater A	Leave one session out	530-631	75.9-79.2%	0.449-0.583
rule output vs rater B	Primary saved offsets	661	77.8%	0.529033
rule output vs rater B	S021 lookup -3 s	660	77.7%	0.528759
rule output vs rater B	Leave one session out	530-631	76.3-79.2%	0.472-0.571

Only the S021 machine lookup is shifted; the human labels are unchanged. One machine-empty bin is omitted, leaving 660. Exact primary/shifted kappas are 0.530413/0.530126 for rater A and 0.529033/0.528759 for rater B. Similar rounded estimates do not show that bin support or historical alignment is identical.

Table S11. Complete three-group agreement count matrices.

Comparison	Row label	Column: low	Column: ordinary	Column: rapid
inter-rater A vs B	low head motion	416	23	7
inter-rater A vs B	ordinary head motion	44	125	2
inter-rater A vs B	rapid head motion	2	14	28
rule output vs rater A	low head motion	370	70	6
rule output vs rater A	ordinary head motion	49	115	7
rule output vs rater A	rapid head motion	9	9	26
rule output vs rater B	low head motion	383	76	3
rule output vs rater B	ordinary head motion	35	111	16
rule output vs rater B	rapid head motion	10	7	20

For A versus B, rows are A and columns B. For rule versus rater, rows are the named rater and columns the separate three-group output. Each matrix contains 661 bins. Agreements are 569/661, 511/661, and 514/661. Point kappas are 0.698262, 0.530413, and 0.529033. Main Table 5 reports the 5,000-resample session-cluster intervals rerun from the original paired returns.

Table S12. Exploratory six-output agreement count matrices.

Comparison	Row label	Rapid	Smooth	Moderate	Excursion	Low	Residual
inter-rater A vs B	Rapid turn	28	0	14	0	2	0
inter-rater A vs B	Smooth turn	0	0	0	0	0	0
inter-rater A vs B	Moderate turn	2	0	125	0	44	0
inter-rater A vs B	Acceleration excursion	0	0	0	0	0	0
inter-rater A vs B	Low motion	7	0	22	0	365	14
inter-rater A vs B	Residual output	0	0	1	0	33	4
rule output vs rater A	Rapid turn	26	0	7	0	0	11
rule output vs rater A	Smooth turn	0	0	0	0	0	0
rule output vs rater A	Moderate turn	7	0	97	0	0	67
rule output vs rater A	Acceleration excursion	0	0	0	0	0	0
rule output vs rater A	Low motion	6	0	24	0	0	378
rule output vs rater A	Residual output	0	0	4	0	0	34
rule output vs rater B	Rapid turn	20	0	5	0	0	12
rule output vs rater B	Smooth turn	0	0	0	0	0	0
rule output vs rater B	Moderate turn	16	0	95	0	0	51

Comparison	Row label	Rapid	Smooth	Moderate	Excursion	Low	Residual
rule output vs rater B	Acceleration excursion	0	0	0	0	0	0
rule output vs rater B	Low motion	3	0	32	0	0	409
rule output vs rater B	Residual output	0	0	0	0	0	18

Rows represent the first/named human reference; columns represent the comparison source. Human labels are mapped as in Table S5. This exploratory mapping is not the primary three-group rule and does not establish a complete behavioral ground truth. Zero-support reference classes are not evidence of perfect detection.

Table S13. Adjacent exported-sample output counts by timing group.

Group	From / to	Rapid	Smooth	Moderate	Excursion	Low	Residual
ADB-like	Rapid turn	1,089 (65.3%)	0 (0.0%)	452 (27.1%)	8 (0.5%)	0 (0.0%)	118 (7.1%)
ADB-like	Smooth turn	-	-	-	-	-	-
ADB-like	Moderate turn	414 (4.6%)	0 (0.0%)	5,513 (61.6%)	182 (2.0%)	0 (0.0%)	2,845 (31.8%)
ADB-like	Acceleration excursion	14 (3.2%)	0 (0.0%)	130 (29.5%)	60 (13.6%)	0 (0.0%)	237 (53.7%)
ADB-like	Low motion	-	-	-	-	-	-
ADB-like	Residual output	149 (0.5%)	0 (0.0%)	2,852 (9.9%)	191 (0.7%)	0 (0.0%)	25,596 (88.9%)
browser-compatible	Rapid turn	23 (48.9%)	4 (8.5%)	17 (36.2%)	0 (0.0%)	0 (0.0%)	3 (6.4%)
browser-compatible	Smooth turn	5 (0.5%)	837 (83.6%)	0 (0.0%)	17 (1.7%)	0 (0.0%)	142 (14.2%)
browser-compatible	Moderate turn	15 (2.7%)	2 (0.4%)	330 (60.2%)	29 (5.3%)	0 (0.0%)	172 (31.4%)
browser-compatible	Acceleration excursion	1 (1.2%)	12 (14.3%)	21 (25.0%)	8 (9.5%)	0 (0.0%)	42 (50.0%)
browser-compatible	Low motion	0 (0.0%)	1 (1.1%)	2 (2.2%)	0 (0.0%)	79 (85.9%)	10 (10.9%)
browser-compatible	Residual output	3 (0.1%)	146 (4.0%)	178 (4.9%)	30 (0.8%)	12 (0.3%)	3,298 (89.9%)

Cells are counts (row percentages); a dash indicates zero row support. The two timing-group panels are retained; their aggregate is available in transition_matrix.csv rather than repeated here. Excluding long-gap boundaries leaves 45,560 - 29 - 242 = 45,289 pairs. Changed-pair fractions are 18.7% overall, 19.1% for ADB-like, and 15.9% for browser-compatible files. These are adjacent software outputs, not behavioral Markov probabilities.
